# Striving towards true equity in global health: A checklist for bilateral research partnerships

**DOI:** 10.1371/journal.pgph.0001418

**Published:** 2023-01-18

**Authors:** Daniel Z. Hodson, Yannick Mbarga Etoundi, Sunil Parikh, Yap Boum

**Affiliations:** 1 Yale School of Medicine, New Haven, CT, United States of America; 2 Douala Military Hospital, Douala, Cameroon; 3 Faculty of Medicine and Pharmaceutical Sciences, University of Douala, Douala, Cameroon; 4 Department of Epidemiology of Microbial Diseases, Yale School of Public Health, New Haven, CT, United States of America; 5 Epicentre, Yaoundé, Cameroon; 6 Faculty of Medicine and Biomedical Science, University of Yaoundé I, Yaoundé, Cameroon; 7 Institut Pasteur of Bangui, Bangui, Central African Republic; Global Malaria Programme, World Health Organization, SWITZERLAND

## Abstract

Interest in “global health” among schools of medicine, public health, and other health disciplines in high-income countries (HIC) continues to rise. Persistent power imbalances, racism, and maintenance of colonialism/neocolonialism plague global health efforts, including global health scholarship. Scholarly projects conducted in low- and middle-income countries (LMIC) by trainees at these schools in HIC often exacerbate these problems. Drawing on published literature and shared experiences, we review key inequalities within each phase of research, from design through implementation and analysis/dissemination, and make concrete and practical recommendations to improve equity at each stage. Key problems facing global health scholarship include HIC-centric nature of global health organizations, paucity of funding directly available for LMIC investigators and trainees, misplaced emphasis on HIC selected issues rather than local solutions to local problems, the dominance of English language in the scientific literature, and exploitation of LMIC team members. Four key principles lie at the foundation of all our recommendations: 1) seek locally derived and relevant solutions to global health issues, 2) create paired collaborations between HIC and LMIC institutions at all levels of training, 3) provide funding for both HIC and LMIC team members, 4) assign clear roles and responsibilities to value, leverage, and share the strengths of all team members. When funding for global health research is predicated upon more ethical and equitable collaborations, the nature of global health collaborations will evolve to be more ethical and equitable. Therefore, we propose the Douala Equity Checklist as a 20-item tool HIC and LMIC institutions can use throughout the conduct of global health projects to ensure more equitable collaborations.

## Introduction

*“No one size fits all approach will succeed within a continent as diverse as Africa…*. *Local solutions should ensure COVID 19 is not only a challenge that is met*, *but also an opportunity to strengthen health systems before the next pandemic*.*”* – Yap Boum II, Lisa M. Bebell, Anne-Cécile Zoung-Kanyi Bisseck [1]

*“[I]f the academic literature to which we give priority does not reflect that local experts are at the forefront of addressing local problems*, *then there is something deeply wrong with that literature*, *because it does not reflect reality*.”–Seye Abimbola [2]

Interest in “global health” among schools of medicine, public health, and other health disciplines in high-income countries (HIC) continues to rise. The COVID-19 pandemic only increased this interest, while at the same time highlighting the interconnectedness of health and disease across political and geographic boundaries and exacerbating current disparities in both health care outcomes and inequities in global health scholarship [3, 4]. Indeed, in 2021 a leading journal solicited an entire collection of responses to the question, “What is Wrong with Global Health”? [5–21]. Persistent power imbalances [4], racism [22], and maintenance of colonialism/neocolonialism [23] plague global health efforts, including global health scholarship. A long litany of concrete manifestations of these fundamental problems includes the issues identified in **Table 1**.

**Table 1:** Current problems with global health research and scholarship.

Elitism and racial homogeneity of global health communities in HIC [11, 14, 57, 61]
Study priorities and design dictated by HIC institutions [4, 8, 17, 43, 58, 61, 79]
Failure to achieve common understanding of the goals of the project [43]
Imbalance of financial resources between LMIC and HIC institutions and paucity of funding opportunities in and from LMIC [13, 43, 60, 61, 77]
Fewer opportunities for mentorship and collaboration in LMIC [9, 77]
Dominance of the English language in the scientific literature [6, 73] and immodest assumptions that the English language literature represents “the sum of all available knowledge” [15]
Problematic language surrounding global health collaborations [6]
Publications prioritized among HIC institutions, while LMIC institutions may not have the same culture or opportunity to research and publish in international peer-reviewed journals
Paucity of training for LMIC investigators and trainees in manuscript preparation
Financial barriers to publication [7, 8, 16, 80]
Absence of benefit or compensation for participants in research studies
Absence of concrete benefits of research output for many LMIC team members
Exploitation of LMIC investigators by using their work for the benefit of HIC investigators without tangible benefit for LMIC team members [79]
Overvaluing quantity over ethical quality of research endeavors [12]
Drawing resources and professionals away from local roles and responsibilities in favor of dedicating time to global health research activities [34]
Drawing LMIC professionals away from their host countries towards long-term careers in HIC settings [77]

Scholarly projects conducted by trainees at schools of medicine, public health, and other health disciplines in HIC often exacerbate these problems, so there has been a reinvigorated discourse to address the principles, processes, and protocols of these institutions [24–30]. HIC institutions must understand that engaging in global health research is not necessarily benign; failing to create equitable partnerships, regardless of original intentions, exacerbates existing structural violence and inequities and causes further harm [31]. At the same time, the training of health professionals constitutes a formative period and pedagogical opportunity to instill in trainees an equitable process for conducting research affecting low- and middle-income countries (LMIC). As such, LMIC institutions must assert the right to challenge and restructure projects to better reflect their needs. We acknowledge the terms HIC and LMIC are themselves highly problematic [6, 7], but we retain them here to draw attention to the unavoidable and often uncomfortable financial disparities that exist between, for example, an Ivy League university in the United States and a public university in sub-Saharan Africa.

Not surprisingly, there have been many different iterations of principles, guidelines, and recommendations to promote more ethical and equitable global health partnerships in development, scholarship, and health care [32–47]. Important recommendations are summarized in **Table 2**. These efforts offer useful theoretical frameworks and guiding questions, although previous iterations have two main limitations. First, participation of LMIC investigators in developing these recommendations has not been optimal. A notable exception is The Global Health Decolonization Movement in Africa, an African initiated and led effort that provides concrete recommendations for global organizations, individual practitioners, funding agencies, academic and training institutions, academic journals, the media, and event organizers [30]. Second, previous efforts often lacked practical steps that can be used during day-to-day decision making and implementation of global health partnerships. Notable exceptions include the toolkits designed by Afsana and colleagues [35] and Pratt [45, 47]. Monette and colleagues state plainly, “there is a general lack of guidance available on how these principles can and should be integrated into practice” [37].

**Table 2:** Existing recommendations for improving equity in global health collaborations.

Authors	Key Points and Recommendations
*Netherlands Development Assistance Research Council (2001) [32]*	• Trust is built over long-lasting partnerships • Capacity strengthening should be an aim of such partnerships • HIC institutions need to “release control and accept considerable autonomy” of LMIC partners
*Swiss Commission for Research Partnerships with Developing Countries (2018) [33]*	• Research questions and methods should be determined “jointly” • Clarify roles and responsibilities of each partner in the collaboration • Be accountable to intended beneficiaries • “Promote mutual learning” and “foster capacit[y] of all parties” • Share data and resources • Disseminate results in ways that allow intended communities to be able to access, understand, and use the results
*Costello and Zumla (2000) [34]*	• Understand that “foreign-led research” can have “negative effects on partner countries” • Encourage “mutual trust and shared decision making” • Emphasize national ownership • Get research findings into “policy and practice” • Strengthen LMIC national research capacity • Equity “needs monitoring by funding agencies”
*Canadian Coalition for Global Health Research (2009, 2019) [35, 42]* *Murphy et al. (2015) [36]*	• Identify goals and “worst-case scenarios” for the partnership for each party • Formalize documents to guide the collaboration (e.g. Memorandum of Understanding, Terms of Reference) • Clearly define roles and responsibilities • Involve all partners in the design phase • Ensure “regular and effective” communication among all parties • Formalize a plan for dissemination of results • Discuss authorship and publication at the outset of the collaboration • Funding agencies “provide critical infrastructure” • Initiate discussion the closure of the partnership during the initial design phase • See the Murphy et al. article for a summary of older sets of principles from 1998–2000
*Monette et al. (2021) [37]*	• Reviewed nine sets of principles for global health research partnerships, yet only four of the nine had authors from LMIC • Summarized the following themes, such as “mutual benefit, agenda setting, equity, accountability, capacity building, sustainability, defining roles, engaging stakeholders, understanding the context, actionable research, communication, data access, humility, inclusivity, mutual learning, social justice, transparency, trust, redress hierarchies, respect diversity of knowledge and skills”
*Bill & Melinda Gates Foundation (2011) [38]*	• Share data broadly and promptly as possible • Respect “identity, privacy, and confidentiality” • Keep “all processes and procedures for data access…transparent, clear” • All team members should remain responsible for the data • “Needs of investigators…must be balanced against those of communities and sponsors that expect health benefits to arise from the activities to which they contribute” • “Aim of benefiting the individuals and communities who enable support inquiry should be furthered to the extent possible and is of particular importance when involving individuals and communities from [LMIC]”
*Centre for Global Health, Trinity College Dublin (2016) [39]*	• “Global health partnerships are dependent on successful relationships” • “Trust was identified by all participants as a prerequisite” • Identify “well defined common objectives and shared benefits” • Ensure “knowledge exchange” and “skills generation” for all partners • Share resources adequately • Respect different capacities of different partners • Promote transparency of communication • Delegate and define roles and responsibilities • From the outset of the collaboration, consider “mediation and conflict resolution” strategies as well as “exit strategies”
*Raza(2005) [40]*	• Effective communication “should be established at all levels” • Each team member should identify their “contribution to the main goals of the collaborative project” • Determine “clear roles of each collaborating team member” • Develop a “tentative timetable for different activities until the termination of the project” • Agree upon “data ownership, keeping, sharing, disclosure and publication” of results • Discuss and assign authorship of publications • Proactively resolve conflicts within the team
*Global Health Task Force of the American Board of Pediatrics (2017) [41]*	• Include diverse perspectives among the leadership • “Sustained engagement results in better understanding and trust” • “Reciprocal and mutually beneficial relationship is a core component of successful partnership” • “A dynamic memorandum of understanding that evolves with [global health] partnership maturity is essential for transparency and trust between partners” • Take steps to “minimize adverse outcomes to visiting providers, students, and trainees as well as to patients, communities, local providers, and health facilities in LMIC” • Assess the “true costs of the program to all institutions involved in the partnership” • “Ensure appropriate reimbursement and …alleviate any undue burden” • “Pretravel preparation for trainees and faculty may improve communication” • “Transparent, open, honest, and unambiguous communication strategy between partners build trust” • “Partnerships are expected to encounter difficulties”, so “conflict resolution and mediation” are key • Regularly evaluate “the extent to which the partnership’s objectives are being achieved”
*Rethink Research Collaborative (2018) [43]*	• Allow time to nurture personal connections and cultivate networking opportunities for all team members • In addition to personal relationships, “[build] on existing networks” and gain “institutional buy-in” • Consider “joint north-south PI model” as one effective structure • “Understand and respond to the ultimate beneficiaries of the research” • LMIC team members require “more funded time” as “insufficient time and funding” and unequal access to funding opportunities remain fundamental barriers to participation for LMIC partners • “Electronic tools and remove/web-based servers” can be used to promote collaboration across geographies • Transparency in budgeting is important • Provide all team members opportunities for professional networking • Focus on “locally defined needs, priorities and practices at all stages” • Ensure “clarity and transparency of …roles/responsibilities” • Promote equitable ownership of data • “Make collaboration at the application stage mandatory….to ensure that [LMIC] partners are aware of the project and the commitment expected”
*Nakanjako et al.(2021) [44]*	• Remember “partnerships form because one organization or group is not able to accomplish something on its own” • Agree on shared values and goals • Build a “true partnership” involving “joint ownership of the program or activity and each partner contributes and adds value in a unique and equitable way” • “Funders have a major role to play in building equitable and effective partnerships” • “Ensure equal participation and decision-making” for LMIC partners • “Maintain regular communications and interim progress reports” • Avoid significant logistical demands on local institutions, such as “local proposal review of projects, demand for office and desk space, overwhelming laboratory services with equipment that need electricity”
*Pratt* (2021) *[45, 47]*	• Assess the ability of the partnership to improve the condition of local communities • Identify how LMIC community members will participate in priority-setting and identify potential barriers to power sharing among partners • Ensure inclusion of perspectives from the “disadvantaged, less influential, lower status, and/or marginalized” • Ensure engagement of the wider community who stands to be involved/benefit from the proposed projects • Empower community members as researchers during design phase • Proactively identify potential “unintended harms” • Research teams should be accountable to the wider community
*Global Health Decolonization Movement in Africa (*2021) *[46]*	• Improve diversity, equity, and inclusion within your organization • Recruit and promote LMIC professionals • Allow LMIC team members influence over “strategic decisions and resources” • Respect, value, and prioritize LMIC expertise • Break negative stereotypes of LMIC and LMIC team members • Champion LMIC trainees and mentees • Reject parachute research proposals • Create curricula to critically evaluate colonial history and its relevance to global health • Be aware of anti-LMIC biases of other actors, such as academic journals, fundings sources, or businesses • Ensure LMIC participations in national/international conferences and events

## The Douala Equity Checklist is targeted for use by *both* HIC and LMIC institutions and investigators

In settings as diverse as a primary care office visit or a hospital operating room, medical professionals use checklists to ensure crucial considerations are not overlooked. Earlier this year, Brunette mused about the possibility of “internal checklists for health equity” [48]. We add to the existing literature by proposing an “equity checklist” for evaluating global health research projects as part of their funding screening and application process. Our recommendations are derived from years or lifetimes of living, working, and learning in LMIC and from almost a decade of jointly attempting to build more equitable collaborations between institutions in the United States and institutions in countries including Uganda [49–51], Burkina Faso [52, 53], Guinea [54], and most recently Cameroon (forthcoming).

**Fig 1:**
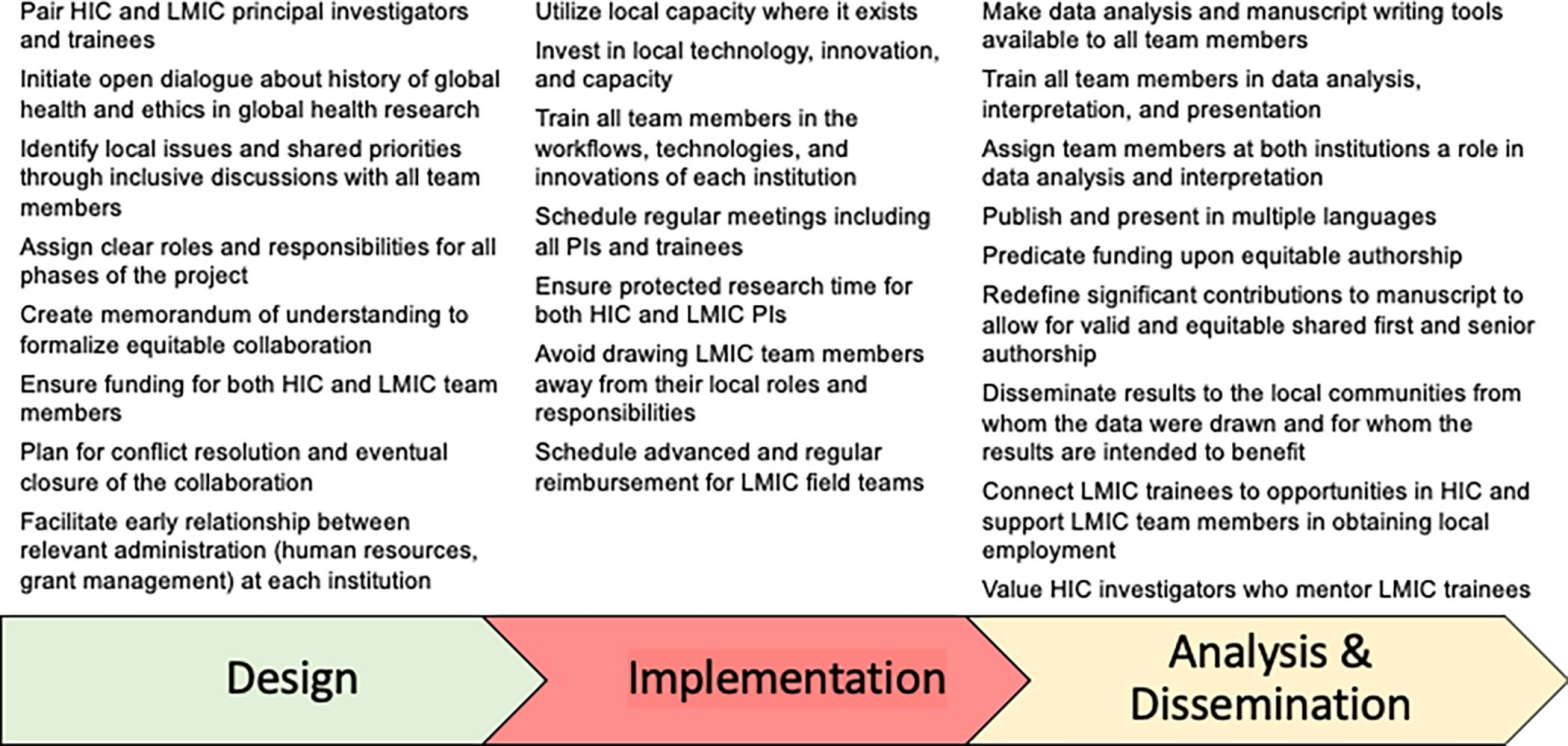
Recommendations for advancing equity in global health during each phase of research.

Four foundational principles underlie all concrete recommendations. First, collaborators should prioritize locally derived and relevant solutions to global health issues. Second, collaborations should ideally be paired between HIC and LMIC at as many levels as possible (i.e. principal investigators, field teams, laboratory staff, trainees). Third, the budgets should provide for paired funding to investigators from both HIC and LMIC countries (e.g. bidirectional travel, conference attendance, dedicated research time). Fourth, collaborations should mutually assign clear roles and responsibilities which value, leverage, and share the strengths of all team members and institutions. These contributions must then be appropriately during the dissemination phase. Guided by these initial principles, we consider each step of the research process similar to Afsana and colleagues [35], first presenting challenges to equity encountered at each step followed by proposed solutions (**Fig 1**). Finally, we present the Douala Equity Checklist (**S1 File**) for those engaging in global health scholarship.

Our recommendations are targeted towards two groups: HIC training institutions involved in global health work and awarding grants to their trainees, and LMIC institutions engaging in collaborations with HIC institutions. On one hand, HIC institutions hold a disproportionate share of power in global health research partnerships; therefore we believe these institutions have an obligation to take concrete anti-colonialist and anti-racist steps to dismantle imbalances of power and to promote equity. HIC institutions need to learn the “lifelong practice” of “allyship”, that is “building relationships based on trust, consistency, and accountability with marginalized individuals and/or groups” [55], and overhauling the way they fund their trainees can be a critical first step in this practice. On the other hand, LMIC investigators and team members should not be silent and passive actors in global health collaborations. We acknowledge the struggle of LMIC investigators and trainees to navigate the chasms of inequities that lie all along the paths of potential collaborations. We encourage LMIC institutions to decide on local issues, solutions, and goals; to determine the terms of global collaborations in which they participate; to decide how global health scholarship involving their institutions should take place; and ultimately to decide whether any given collaboration should occur at all. This checklist may therefore help LMIC team members to identify inequitable collaborations and to provide the grounds on which to reject or improve these collaborations. An initial list of considerations specifically for LMIC investigators considering collaborations with investigators from HIC was also recently proposed [20]. Finally, while we structure this checklist based on trainee-level academic-based projects, we note that the principles set forth apply to global health research across levels and settings.

## The design phase should prioritize local solutions to global health challenges

Historically, the field of global health unfortunately emerged from colonialism. The design phase offers the opportunity for collaborators to learn and share their experiences both about the colonialist origins and harms of global health as well as global health success stories. Unfortunately, attempts at these discussions are often been absent or fail to achieve their ideals [31]. Even recently, the discourse and agenda of global health collaborations continues to be driven by HIC institutions. For example, the overwhelming majority of Master’s programs in “global health” are at HIC institutions and financially inaccessible to most LMIC trainees [56]. Furthermore, the vast majority of “global health” organizations are based in HIC and led by individuals who are from and/or trained in HIC [57]. Finally, HIC team members usually drive the research questions, hypotheses, and conclusions due to increased access to higher education and funding, while LMIC investigators are often relegated to collecting data and coordinating field teams [19, 58, 59]. The stark disparities in funding remain a primary barrier to equity: HIC investigators have access to competitive grants from national and international scientific societies, and many HIC institutions provide minimally competitive, short-term funding for trainee research projects. Such funding may cover visa and travel costs, a living stipend, and conference funding. In contrast, LMIC investigators rarely are equitably represented in awarded funds, and funding opportunities may consist of a short list of highly competitive international grants. This inequity tips the balance of agency and power in favor of HIC researchers. As discussed below, the primacy of English in academic literature places non-English speakers at a marked disadvantage, which is manifest at this stage by their constrained ability to compete for and win grants in the language in which they are proficient and to significantly contribute to the dissemination of the research outcomes [60].

To address inadequacies in team members’ understanding of the field of global health that can exacerbate inequities, all collaborators should formally discuss the field of global health before the project implementation. Important considerations include emphasizing the history of colonization in global health and steps toward decolonization, emphasizing scholarship from LMIC, and even using popular and social media sources as part of the curriculum [31]. All team members should be encouraged to share their previous experiences and lessons learned.

To elevate the voices and agendas of LMIC institutions, establishing paired collaborations is key at all stages. Principal Investigators (PIs) from the HIC institution and the LMIC institution should be paired together as co-PIs, and HIC trainees should be paired with LMIC trainees at similar levels of education and training. The career stages of each PI should be taken into consideration, and we advocate for explicit consideration of potential dynamics and their implications at the outset of the study design phase to maximize academic growth and to avoid tensions that may develop during project execution. For example, arrangements that pull senior LMIC investigators away from mentoring their own trainees or put the HIC and LMIC investigators in direct competition should be avoided. Trust and bidirectional capacity building take time to cultivate, and these collaborations remain a dynamic work in progress.

With paired collaborations, the overarching goals and specific aims of the research projects can arise from bilateral discussions, rather than HIC-centric views of supposed “high-priority” questions needing to be answered. The end goal should be to provide local solutions to local issues that can be eventually stewarded, owned, and sustained by the LMIC institution itself. Specific tools, such as the checklist proposed by Costello and Zumla [34], the Partnership Assessment Toolkit by Afsana and colleagues [35], or the ethical toolkit designed by Pratt [45, 47] may help guide these initial conversations between potential research partners. In addition, all team members require access to academic journals and unpublished research to allow equal access to the most up-to-date literature. Finally, we agree with the importance of a memorandum of understanding (MOU) that “commits each partner to transparency in all aspects of the project administration and budgeting; and that sets out clearly the right of all partners regarding acknowledgement, authorship, intellectual property and data use” [43] and importantly “outline[s] who does what, specifying the roles and responsibilities of the partners” [59].

To address inequities in funding, we propose three solutions. First, when HIC institutions award their own funding opportunities, these institutions need to design the budgets to be more equitable, and we specifically challenge HIC institutions to adapt their global health grants to provide direct funding for both HIC and LMIC trainees for all the activities historically available only to HIC trainees, such as 1) visiting the other country, 2) receiving a living stipend, 3) traveling to national or international conferences, and 4) disseminating the results via presentations and publications.

Second, HIC institutions and trainees need to accept “fewer seats at the table” [55], and we challenge HIC institutions to create mechanisms to directly fund LMIC trainees, investigators, and institutions [61]. We recognize that this may result in fewer grants and fewer trainees from HIC institutions conducting global health research, but we believe a lower number of more equitable collaborations would prove more sustainable in the long-term. As Lioba A. Hirsch passionately and eloquently argues,

“If global health institutions are serious about their commitment to working against the legacies of colonialism and fighting racism, then they will need to give up some or all of their power. That means a radical redistribution of funding away from high-income countries, a loss of epistemic and political authority, and a limitation to our power to intervene in [LMIC]” [22].

Thirdly, we encourage the growing cadre of LMIC upper class and philanthropists to create and maintain funding mechanisms for LMIC trainees engaged in global health scholarship [59].

## Building sustainable capacity in all aspects of research implementation is critical if equity is to be achieved in the long-term

Too often during the implementation phase, samples or data are viewed as another resource to simply be extracted from LMIC settings, as HIC trainees “parachute” into the LMIC, recruit patients, obtain samples, create data, and then leave with no intention of establishing long-term collaborations or long-term sharing of the samples or data [62]. Furthermore, investigators at HIC institutions often have protected (i.e. funded) research time, while LMIC team members too often rely on day-to-day income generation. As such, financial necessity may limit the time LMIC team members are able to dedicate to the research project. Furthermore, while team members such as lab technicians and research coordinators enjoy salaried positions at HIC institutions, their counterparts in LMIC often lack a regular income. Field workers make irreplaceable contributions to the research efforts, yet failure to provide timely compensation in LMIC can leave these crucial collaborators unable to meet their basic, daily needs.

To avoid so-called parachute research, analyses should be performed locally when local capacity exists, and if absent, efforts for bilateral training/capacity building should be included as part of the study design. In our work in infectious diseases, for example, some analyses must be performed in the local setting by necessity (e.g. rapid diagnostic tests), some clearly lend themselves to significantly better analysis locally (e.g. microscopy), while others may be performed in either the HIC or LMIC (e.g. molecular-based diagnostic testing). Importantly, LMIC teams may have a methodology or technology that is superior to that available in HICs, but too often, innovations from LMIC are ignored to the determent of scientific progress. For example, many studies explore the possibility of anti-*Plasmodium* activity of plant-based compounds in sub-Saharan Africa [63–65]. but the resources to fully investigate their clinical utility remains limited to a few African settings.

Furthermore, all PIs and trainees should be actively involved in issues surrounding participant recruitment and sample procurement. Increasing utilization of digital communication platforms during the COVID-19 pandemic has highlighted opportunities for collaborators from around the globe to meet regularly using phone or video calls. Geographic distance should no longer present a barrier to active communication among team members.

To partially address technology and capacity gaps between countries and institutions, each institution should host investigators/trainees from the other country to provide exposure and training in their workflows, technologies, and innovations as these are unique to each local setting. Finally, we challenge HIC institutions to provide financial support to enable development and usage of new technologies at LMIC institutions. HIC team members gain professional training and advancement through their global health research, so LMIC collaborators should correspondingly expect to develop new skills and improve their overall quality of life.

To address inequities in dedicated project time, funded research time for all team members is essential during the implementation phase. For senior investigators, HIC and LMIC institutions need to work towards ensuring protected research time for the LMIC principal investigator. In some cases, it may be necessary for funding from the HIC institution to directly provide for this protected time. However, in doing so, the caution of Costello and Zumla from two decades ago remains true: research partnerships should avoid stealing LMIC professionals away from their local responsibilities [34]. For LMIC field teams, funding should provide an advance at the start of research and regular and scheduled payments during the project for field teams to allow them to meet their daily expenses and needs.

## LMIC partners should ultimately drive data analysis and dissemination of locally conducted research

Too often, data collection occurs in LMIC while data analysis occurs in HIC settings, followed by a dissemination process which itself provides a poignant and highly quantifiable example of the disadvantages faced by LMIC investigators. Publication in scientific journals is almost universally a high priority for HIC academics, but LMIC team members have less opportunity to publish in international journals, and this can lead HIC team members to incorrectly perceive that there is less importance for LMIC team members to publish. Local contributors from LMIC are also less likely than their HIC collaborators to attend global conferences [66] or to be listed among the authorship on published manuscripts, especially in either coveted first or senior author positions [67–71]. Especially damning, one study focusing on maternal health found a dose-response relationship between the (decreasing) income level of the host country and the (decreasing) proportion of articles with a local first author [70].

For many LMIC team members, language remains a major barrier to dissemination at the international level. English may be the dominant language of scientific literature, but it is certainly not the primary language in the cities and villages where global health work is conducted. For example, review of each African country’s official languages reveals that only 46% even consider English an official language [72], and colonial languages do not reflect the thousands of local languages spoken across communities on the continent. Publishing solely in English can ostracize non-English speaking team members and prevent them from fully engaging in the publication process or even from interpreting the definitive, published results [60, 73]. The English language scientific literature cannot and does not reflect all of civilization’s knowledge on any topic. As Himani Bhakuni and Seye Abimbola brilliantly state:

“In another example of credibility deficit related to gaze or audience, researchers can justify a study or publication on the basis of a gap in the literature, as if the literature could be considered the sum of all available knowledge” [15].

Furthermore, collaborators should remain aware that presenting and publishing in multiple colonial languages does not address dissemination of results to field teams who may use local languages and may have limited competency in colonial languages. These team members who were essential to the implementation phase may lack access to the scientific literature in any language.

To address inequities in data analysis and interpretation, several changes are needed. Resources such as statistical software and consultants, trainings on manuscript writing, and scientific proofreading services must be made available to all team members. Team members from both HIC and LMIC should be assigned roles in data analysis and interpretation. Active involvement of all team members in this phase should be expected, and virtual meetings to share and discuss results, data analysis, and interpretation of preliminary results may be a more inclusive medium than simply sharing comments on a manuscript [74, 75].

To address the English language bias in publication, we propose several solutions. First, journals need to expand the space for publication of entire manuscripts (not only abstracts) in languages other than English [73, 76], and investigators should strive to publish the same data in multiple languages. Journals should provide interpreter services to allow entire manuscripts to be published in multiple languages in the same journal in order to reach different audiences. Investigators should also consider publishing in local journals, such as the health journals listed on African Journals Online (https://www.ajol.info/index.php/ajol/browseBy/category?categoryId=17). Second, scientific conferences should provide live interpreter services to allow non-English speakers to present their results in a language more comfortable for them and to better understand sessions presented in English.

To address inequities in authorship, we propose two concrete solutions. First, funding from HIC institutions should be predicated upon shared authorship, such as the trainee from one institution serving as first author under the mentorship of the senior investigator from the other institution or co-first and co-last authorship. While it is an easy gesture to add a collaborator to a manuscript, the true task is to empower, engage, and support collaborators from both institutions to work through the manuscript submission process. Authorship should always reflect true contributions on all sides of the collaboration, but common interpretation of the International Committee of Medical Journal Editors (ICJME) criteria for authorship may not reflect the range of critical contributions from all colleagues, especially contributions from LMIC team members [75]. Achieving equity in co-written and co-produced academic works (posters, presentations, manuscripts) may require reframing the manner in which contributions are defined; for example, including oral, rather than written, feedback [74, 75]. Second, HIC institutions and their promotion review committees also need to reframe their expectations and values: investigators should be rewarded not only for first or senior author publications, but also for working with LMIC students and investigators to help them achieve the same [59, 77].

To address the failures to disseminate results to the communities from whom the data were drawn or for whom the results were intended to benefit, dissemination of results should occur across multiple media, including scientific journals, presentations at each institution in the HIC and LMIC, and local media that are accessible to all collaborators and the communities from which participants/samples were drawn [31]. In addition to publishing the results in a peer-reviewed journal, the team should ask, how can these results also be presented in a locally digestible and impactful format? Such media may include radio, television, social media, and in-person presentations at traditional gatherings or places of worship; all avenues that LMIC team members are in the best position to access.

To address inequities in which team members benefit from the collaboration, we challenge HIC institutions to create pathways for LMIC team members to participate in future trainings and degree programs at the HIC institution. Such opportunities may take the form of in-person or virtual short-term degree or certificate programs provided to LMIC trainees, and the pandemic has highlighted the power of virtual classroom formats to deliver training to geographically distant trainees. HIC team members and institutions can also easily support LMIC team members in obtaining quality local employment, for example by providing letters of recommendation.

## Conclusions

The Douala Equity Checklist (**S1 File**) provides a practical tool for both HIC and LMIC institutions and researchers to evaluate the equity of collaborations in global health. Proposals that do not reasonably address these key equity issues should not be funded by HIC institutions, should be rejected by LMIC institutions, and if applicable, be revamped. ***When funding for global health research is predicated upon more ethical and equitable collaborations*, *the nature of global health collaborations will evolve to be more ethical and equitable*.** We believe the foundational principles outlined in our recommendations can extend to collaborations of any magnitude, and national and international donors awarding larger scale grants to investigators across institutions need to prioritize *direct* funding to LMIC investigators, especially for issues affecting LMIC. Recommendations for equity in such large scale funding processes have recently been published [61].

COVID-19 and our growing interconnectedness have led to ever greater interest in “global health” among HIC health professional schools. Academia needs to constantly reevaluate the manner in which global health scholarship is conducted. Too often, global health flows along the all-too-well established fault lines of colonial relationships, as samples and data are simply extracted from host countries and exploited by HIC collaborators for their professional and personal gain. As many HIC institutions devote more financial and human resources to their global health interest groups, global health office administration, and DEI (Diversity, Equity, and Inclusion) offices, we recognize that these resources focus mainly on the HIC institutions themselves. To truly support global health efforts and to forge bidirectional global health collaborations between relatively resource-rich HIC institutions and relatively resource-limited LMIC institutions, transformative new models for collaboration, backed with adequate funding, are needed. ***We are not naïve to the major challenges that our recommendations will encounter*, *but new paradigms must be discussed and entertained in order to bring about such transformative change*.** We hope consideration of these points and utilization of the Douala Equity Checklist can transition the field from a dialogue of the problems to implementation of practical solutions, eventually leading to the “promised land” [78] of truly equitable global health collaborations in the years to come.

## Supporting information

S1 File Douala Equity Checklist. (DOCX)
