# Supplementary material for: Striving towards true equity in global health: A checklist for bilateral research partnerships
Source: PLOS Glob Public Health. 2023 Jan 18;3(1):e0001418. doi: 10.1371/journal.pgph.0001418 (PMC10021183; doi:10.1371/journal.pgph.0001418)
Supplement: S1 File — (DOCX) [file pgph.0001418.s001.docx]

**S1 File: Douala Equity Checklist**

| Design Phase | | |
| --- | --- | --- |
| Item Met | **Item** | **Detailed Sub-Items** |
|  | 1. The research team consists of pairs of team members at various stages of training as applicable | - At least one PI from HIC institution and one PI from LMIC institution - At least one trainee from HIC institution and one trainee from LMIC institution |
|  | 1. All team members will receive training/education in the history of global health in several key areas, such as the following: | - Origins of global health including colonial origins and harms - Background in the ethics of global health research - Examples of projects (both successful and failed) in the specific LMIC - Examples of LMIC innovations applied in the HIC |
|  | 1. Investigators and trainees from both institutions have identified and applied for support (may be pecuniary or material) | - HIC team members have applied for funding/support - LMIC team member have applied for funding/support |
|  | 1. When applicable, the grant provides funding for both the HIC trainee and LMIC trainee for all the following: | - Visa, travel expenses, and living stipend for each trainee to travel to the other country - Travel and registration fees for international conferences |
|  | 1. Each trainee will receive training in the skills and techniques necessary for the successful completion of the project. | - At a minimum, each trainee will receive basic formal language training (e.g. classes, tutors) tailored to the countries and topics involved |
|  | 1. A Memorandum of Understanding (MOU) describing the details of this checklist and any other considerations will be written and signed before the project begins. The MOU must address all the following: | - Authorship should be decided *a priori* and allow for each trainee to take the lead and each senior investigator to serve as senior author on different posters/presentations/manuscripts - Trainees and investigators from both HIC and LMIC institutions will have equal access to the samples, data, and results from the project - The process for utilizing the samples, data, and results from this project for other manuscripts and projects will be clearly defined - The process to change or amend the MOU will be clearly defined |
|  | 1. All team members will have access to relevant technological resources, as applicable: | - Communication software (e.g. Zoom, Skype) - Scientific databases (e.g. Scopus, Web of Science, PubMed, Science Direct) - Database software (e.g. REDCap, Qualtrics, OnCore) - Statistical packages (e.g. SPSS, Stata, R, SAS, MATLAB, GraphPad) - Word processing and presentation software (e.g. Microsoft suite, Adobe suite) |
|  | 1. All team members from both HIC and LMIC can identify specific examples of how this project will advance their training/career and/or improve their overall quality of life | - Project has specific benefit to HIC team members - Project has specific benefit to LMIC team members |
| Execution Phase | | |
|  | 1. The HIC and LMIC each host stages of the project. Key considerations include the following: | - When capacity already exists in the LMIC (e.g. to perform certain analyses), that stage of the project will take place in the LMIC - When capacity does not yet exist in the LMIC, the LMIC institution will receive training and supplies to expand its capacity to perform similar research in the future - Team members from both institutions will be trained in the workflows, innovations, and technologies of each institution |
|  | 1. Both PI will have funded, dedicated time to complete the roles and responsibilities of the project | - HIC PI will have protected time dedicated to the project - LMIC PI will have protected time dedicated to the project |

|  | 1. All team members have the time for regular communication for the duration of the project | - All PI commit to regular communication - All trainees commit to regular communication |
| --- | --- | --- |
|  | 1. The local roles and responsibilities of all team members have been explicitly discussed, and all will be able to participate in the project without neglecting their local roles and responsibilities | - Local roles of all PI have been discussed - Local roles of all trainees have been discussed - Local roles of local staff and field workers have been discussed |
|  | 1. LMIC field workers will receive timely, regular, and reasonably compensation. At a minimum, the following will all be met: | - A portion of the compensation will be distributed at the debut of the project - Compensation will occur at clearly defined, regular intervals during the project - Payment will be made directly to field workers themselves (e.g. via electronic payments) rather than through local intermediaries - The HIC institution has sufficient liquid funds available to cover this compensation in the case of delays in the disbursement of this grant |
|  | 1. The health of LMIC field workers will be protected throughout the duration of the project, including all the following: | - LMIC field workers will not be subject to unnecessary dangers in the implementation of the project - The costs of any injury or illness suffered as direct result of the project will be covered by the HIC and LMIC institutions |

| Analysis and Dissemination Phase | | |
| --- | --- | --- |
|  | 1. Trainees from both institutions will have access to training and resources relevant to data analysis and dissemination including the following as applicable: | - Training on statistical methods and access to consultants - Training on preparation of scientific manuscripts - Access to writing and proofreading services |
|  | 1. All trainees will be assigned roles in data analysis and interpretation | - HIC trainees have assigned roles - LMIC trainees have assigned roles |
|  | 1. Results will be disseminated among all involved communities and institutions in both countries including all the following: | - The HIC institution - The LMIC institution - The communities from which participants or samples were drawn |
|  | 1. Results will be disseminated to the above institutions and communities using the language and media most appropriate to each setting, such as the following: | - Manuscripts will be published in a language that is accessible to other professionals from the LMIC - Results will be disseminated in local media easily accessible to local audiences in the LMIC (e.g. TV, radio, newspaper, social media) |
|  | 1. Authorship and future collaborations will proceed according to the MOU, including the following key considerations: | - Authorship on all posters, presentations, and manuscripts will proceed per the MOU with team members from HIC and LMIC alternating key author positions or having formal co-authorship - Criteria for authorship will be evaluated on the basis of overall contribution to the project, data analysis, and dissemination rather than strictly by written contribution to the scientific manuscript - If the data and results will be used for additional manuscripts or projects, all team members will be informed and credited as defined in the MOU |
|  | 1. Team members will be supported in future endeavors | - Trainees from both institutions will be connected to opportunities to pursue further training at either institution - Team members will support LMIC field workers in pursuing additional employment, such as validating their involvement in the project, serving as professional references, or providing letters of recommendation, |
